# Infantile hypertrophic pyloric stenosis at a tertiary care hospital in Tanzania: a surgical experience with 102 patients over a 5-year period

**DOI:** 10.1186/s13104-015-1660-4

**Published:** 2015-11-18

**Authors:** Phillipo L. Chalya, Mange Manyama, Neema M. Kayange, Joseph B. Mabula, Alicia Massenga

**Affiliations:** Department of Surgery, Bugando Medical Centre, Mwanza, Tanzania; Department of Anatomy, Catholic University of Health and Allied Sciences-Bugando, Mwanza, Tanzania; Department of Pediatrics, Catholic University of Health and Allied Sciences-Bugando, Mwanza, Tanzania; Department of Surgery, Catholic University of Health and Allied Sciences-Bugando, Mwanza, Tanzania

**Keywords:** Infantile hypertrophic pyloric stenosis, Treatment, Outcome, Tanzania

## Abstract

**Background:**

Infantile hypertrophic pyloric stenosis (IHPS) is the most common cause of gastric outlet obstruction in infants. There is paucity of published data regarding this condition in our setting. This study describes the clinical presentation, mode of treatment and outcome of treatment of this disease and identifies factors responsible for poor outcome of these patients.

**Methods:**

This was a descriptive retrospective study of infants with HPS admitted to Bugando Medical Centre and subsequently underwent surgery between February 2009 and January 2014.

**Results:**

A total of 102 patients (M:F = 4.7:1) were studied. The median age at presentation was 5 weeks. The median duration of illness was 4 weeks. Fifty-four (52.9 %) patients occur in first-born children. Associated anomalies were reported in 7 (6.9 %) patients. Non-bilious vomiting was the most frequent symptom and it was described in all (100 %) patients. A palpable mass was found in 23.5 % of infants. The diagnosis of IHPS was made clinically in 86 (84.3 %) and by ultrasound in 16 (15.7 %) patients. The treatment was Ramstedt’s pyloromyotomy in all cases. There were 6 (5.9 %) intra-operative mucosal perforations which were repaired successively. Postoperative complication was 11.8 %. The median length of hospital stay was 12 days and it was significantly associated with prolonged pre-operative hospitalization (p = 0.001). The mortality rate was 4.9 %. Age below 2 weeks, late presentation (≥14 days), severe dehydration on admission, hypokalaemia on admission and surgical site infection were the main predictors of mortality (p < 0.001).

**Conclusion:**

This study has shown that IHPS is a common condition in our setting. Age <2 weeks, delayed presentation, prolonged preoperative hospital stay, surgical site infection and high proportion of dehydration and electrolyte disturbance were the main predictors of poor outcome. A high index of suspicion is needed in infants with non-bilious vomiting to avoid delay in diagnosis.

## Background

Infantile hypertrophic pyloric stenosis (IHPS) is a common infantile disorder characterized by enlarged pyloric musculature and gastric outlet obstruction [1]. Historically, it was first described as a disease entity in 1888 by Harald Hirschsprung [2]. IHPS occurs in approximately 1–4 per 1000 live births, although rates and trends vary markedly from region to region [3, 4]. It is more common in males than females (4:1–6:1) [3, 5] and in infants born preterm as compared with those born at term [6–12]. The etiology of IHPS is obscure but probably is multifactorial, involving genetic predisposition and environmental factors [13, 14]. IHPS typically presents with progressive projectile non-bilious vomiting which commences between second and 8th weeks of age [15, 16]. Patients are classically described as being emaciated and dehydrated with a palpable “olive-like” mass at the lateral edge of the rectus abdominus muscle in the right upper quadrant of the abdomen. Peristaltic waves may be seen progressing across the child’s upper abdomen from left to right just before emesis [16].

The diagnosis of IHPS is usually established by abdominal ultrasound, on which IHPS is characterized by increased pyloric muscle thickness length and diameter [17, 18]. Laboratory evaluation classically shows a hypochloremic, hypokalemic metabolic alkalosis resulting from loss of large amounts of gastric hydrochloric acid, the severity of which depends upon the duration of symptoms prior to initial evaluation [19]. Extramucosal pyloromyotomy, introduced by Ramstedt in 1912, still remains the gold standard for surgical management of IHPS [20–26]. The timing of surgery depends upon the clinical status of the infant. If the diagnosis is made early and the child is well-hydrated with normal electrolytes, then the surgery might take place on the day of diagnosis [26]. Surgery should be delayed if there are dehydration and/or electrolyte derangements [27].

The paucity of published data regarding IHPS in most developing countries including Tanzania, prompted the authors to analyze this problem. The study was conducted to describe our experience on the management of infantile hypertrophic pyloric stenosis in our local setting outlining the clinical presentation, mode of treatment and outcome of treatment of this disease and identify factors responsible for poor outcome of these patients. The study intended to highlight challenges associated with the care of these patients and proffer solutions for improved outcome.

## Methods

### Study design and setting

This was a descriptive retrospective study that was conducted in our local setting to describe our experience on the management of infantile hypertrophic pyloric stenosis among infants admitted to the paediatric surgical wards of Bugando Medical Centre (BMC) and subsequently underwent surgical procedure over a period of 5-years between February 2009 and January 2014. Bugando medical centre is the only tertiary health institution serving the whole of the northwestern part of Tanzania, serving a population of about 13 millions. It is a 1000 bed referral hospital located in Mwanza city in the northwestern Tanzania on the southern border of Lake Victoria. It is also a teaching hospital for the Catholic University of Health and Allied Sciences- Bugando (CUHAS- Bugando).

### Study population

The study population included all infants who were admitted to BMC with the diagnosis of IHPS during the period of study and subsequently underwent surgical procedure. Patients with incomplete data were excluded from the study. The diagnosis of IHPS was made clinically by the typical clinical presentation of non-bilious vomiting, gastric peristalsis and palpable pyloric tumor and by abdominal ultrasound on which IHPS is characterized by increased pyloric muscle thickness ≥3 mm and pyloric channel length 14 mm or greater. Dehydration was defined based on the following parameters: sunken fontanelle, dry mucous membranes, poor skin turgor and lethargy The details of patients were retrieved from the patient files kept in the medical record department, paediatric surgical wards and operating theatre. The data collected included age, sex, birth order, associated anomalies, clinical presentation, electrolytes abnormalities on admission, treatment and outcomes of treatment. The outcome variables in this study were postoperative complications, length of hospital stay and mortality. This information was collected using a pre-formed questionnaire.

### Statistical data analysis

The statistical data analysis was performed using statistical package for social sciences (SPSS) version 17.0 for Windows (SPSS, Chicago, IL, U.S.A). The median + interquartile range (IQR) and ranges were calculated for continuous variables whereas proportions and frequency tables were used to summarize categorical variables. Chi-square (χ2) test were used to test for the significance of association between the independent (predictor) and dependent (outcome) variables in the categorical variables. The level of significance was considered as p < 0.05. Study variable that was found to be statistically significant in univariate analysis were subjected to multivariate logistic regression analysis. Multivariate logistic regression analysis was used to determine predictor variables that predict the postoperative complications, hospital stay and mortality.

### Ethical consideration

Ethical approval to conduct the study was obtained from the CUHAS-Bugando/BMC joint institutional ethic review committee before the commencement of the study.

## Results

### Patient’s characteristics

**Fig. 1:**
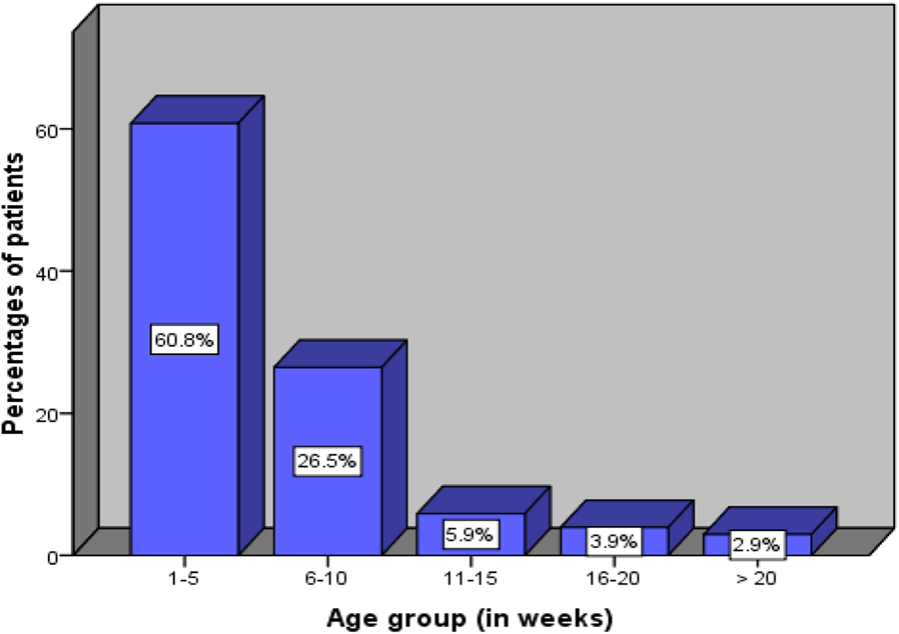
Distribution of patients according to age group (in weeks)

A total of 122 IHPS patients were admitted and operated during the period of study. Among these, 20 were excluded from the study due to incomplete data. Thus, 102 patients all of them of African heritage formed the study population. Out of 102 patients, 84 (82.4 %) were males and 18 (17.6 %) females with a male to female ratio of 4.7:1. The youngest child operated on was 1 week old and the oldest was 26 weeks with the median age of 5 weeks (IQR = 2–6 weeks). The modal age group was 1–5 weeks accounting for 60.8 % of cases (Fig. 1). Fifty-four (52.9 %) patients occur in first-born children. Associated congenital anomalies including neural tube defect (NTD), undescended testis and inguinal hernia in two patients each respectively and recto-vaginal fistula in one patient were reported in 7 (6.9 %) patients. Ninety-six (94.1 %) were breastfed infants and 4 (3.9 %) were bottle fed. Feeding status was not documented in two (2.0 %) patients.

### Clinical presentation

The duration of symptoms before presentation ranged from 1–10 weeks with a median of 4 weeks (IQR of 2–6 weeks). There were 24 (23.5 %) infants who started vomiting very early during the 1st week of life, while the majority of patients, 72 (70.6 %) became symptomatic within 1 month of age. Only 6 (5.9 %) patients became symptomatic after 1 month of age. Progressive non-bilious vomiting was the most frequent symptom and it was described in all (100 %) patients (Table 1).

**Table 1:** Distribution of patients according to clinical presentation

Clinical presentation	Frequency	Percentages
Non-bilious projectile vomiting	102	100
Weight loss	74	72.5
Failure to pass stool	62	60.8
Dehydration	60	58.8
Gastric peristalsis	45	44.1
Palpable mass	24	23.5
Other symptoms	20	19.6

### Diagnosis of infantile hypertrophic pyloric stenosis and laboratory investigations

The diagnosis of Infantile hypertrophic pyloric stenosis was made clinically in infants who presented with a triad of non-bilious projectile vomiting, gastric peristalsis and palpable abdominal mass in 86 (84.3 %) and by abdominal ultrasound in 16 (15.7 %) patients. Other diagnostic investigations such as upper GI endoscopy and barium contrast studies were not performed in any of our patients. Serum electrolytes results were available in 54 (52.9 %) patients and revealed hypokalaemia in 36 (66.7 %) patients, hyponatraemia in 22 (40.7 %) patients and hypochloraemia in 18 (33.3 %) patients.

### Treatment modalities

All patients (100 %) in this study were treated surgically after resuscitation. The median preoperative hospital stay was 8 days (IQR = 6–10 days). The majority of patients presented with the characteristic clinical picture of the surgical condition and all were managed by the standard Fredet-Ramstedt pyloromyotomy. In all patients general anesthesia was used. Supra-umbilical transverse skin-crease incision was used in all cases. There were 6 (5.9 %) intra-operative mucosal perforations which were repaired successively. Post-operatively, test feeds were started within 12 h of surgery in 74 (72.5 %) of the infants and within 12–24 h in 14 (13.7 %) patients. Six (5.9 %) infants who had intra-operative mucosal perforations were kept nil per oral for more than 24 h.

### Treatment outcome and follow up of patients

A total of 22 postoperative complications were recorded in 12 patients giving a complication rate of 11.8 %. Out of the 22 complications, surgical site infection was the most frequent complications accounting for 27.3 % (5/22) of cases (Table 2). All postoperative complications were treated conservatively except wound dehiscence in four patients which required surgical correction. The overall length of hospital stay ranged from 1–14 days with a median of 12 days (+IQR of 10–14 days). Prolonged preoperative length of hospital stay was statistically significantly associated with prolonged overall length of hospital stay (p = 0.001). The median postoperative length of hospital stay was 5 days (QIR = 2–6 days).

**Table 2:** Distribution of patients according to postoperative complication (N = 22)

Postoperative complications	Frequency	Percentages
Surgical site infection	5	27.3
Postoperative vomiting	4	18.2
Wound dehiscence	4	18.2
Pneumonia	3	13.6
Urinary tract infection	3	13.6
Paralytic ileus	1	4.5
Septicemia	1	4.5
Incisional hernia	1	4.5

In this study, five patients died making a mortality rate of 4.9 %. All deaths occurred during the first postoperative day. The causes of death were sepsis, severe dehydration and electrolyte imbalance in one patient each respectively. One patient had unrecognized mucosal perforation later on developed peritonitis and died of cardiac arrest on table during laparotomy for peritonitis. The cause of death was not documented in one patient. According to multivariate logistic regression analysis, age < 2 weeks [OR = 2.74, 95 % CI (1.13–6.44), p = 0.012], late presentation (≥14 days) [OR = 3.92, 95 % CI (1.91–6.94), p = 0.014], severe dehydration on admission [OR = 6.41, 95 % CI (5.11–8.97), p = 0.000], hypokalaemia on admission [OR = 4.63, 95 % CI (1.42–7.91), p = 0.001] and surgical site infection [OR = 4.32, 95 % CI (2.22–8.84), p = 0.003] were the main predictors of mortality. All the 94 survivors in this study were discharged well and no recurrence was reported.

## Discussion

Since it was first described by Harald Hirschsprung in 1888, IHPS has been reported to be a most common cause of gastric outlet obstruction in infancy and the most common surgical cause of vomiting [1, 2]. In this study the males were more affected than females with a male to female ratio of 4.7:1 which is comparable to the global ratio of 4:1–6:1 [3]. The reason for this male predominance is unclear and warrants further investigation. Our study showed higher incidence of IHPS in the first born infants and male sex which is in agreement with most literatures [8–10]. The reason for this observation is not clear. In about 6–33 % of infants with IHPS, associated anomalies have been described in the central nervous system (CNS), gastrointestinal tract (GIT), and urinary tract [16]. In this study, associated anomalies were reported in 6.9 % of cases.

The mean duration of illness in this study of 4 weeks was longer than most published series [28]. This could be due to lack of awareness of families to consider the vomiting as minor symptom and ignore it, or misdiagnosis of the disease. This delayed presentation is a common phenomenon in developing countries and can lead to delay in the diagnosis. Delay in diagnosis can result in significant electrolyte imbalance, weight loss, and failure to thrive [17, 18]. We could not establish the reasons for the late presentation in this study.

The definitive diagnosis of IHPS is usually confirmed by abdominal ultrasound, on which IHPS is characterized by increased pyloric muscle thickness length and diameter. However, in experienced hand, a careful clinical examination provides a definitive diagnosis for most infants with HPS and abdominal ultrasound is reserved for atypical cases [5]. The clinical diagnosis is easily made if the presenting clinical features are typical, with projectile vomiting, visible peristalsis, and a palpable pyloric tumor. In the present study, the diagnosis of IHPS was made clinically in more than 80 % of patients and abdominal ultrasound was employed in only 15.7 % of patients as it was not always readily available in our centre.

In patients with IHPS, serum electrolytes should be measured immediately when the patient arrives in hospital. If vomiting has been ongoing for several days, serum electrolytes are frequently deranged. The nature of derangement is a spectrum, ranging from mild to severe hyponatraemia, hypochloraemia, hypokalaemia, and metabolic alkalosis [17, 18]. In the current study, serum electrolytes results revealed hypokalaemia (66.7 %), hyponatraemia (40.7 %) and hypochloraemia (33.3 %) which is an expected occurrence in untreated cases [17, 18, 29]. This electrolyte derangement in our series can be explained by the fact that the majority of our patients presented late to the hospital when electrolyte imbalance had set in. Prolonged delay in diagnosis can lead to dehydration, poor weight gain, malnutrition, metabolic alterations, and lethargy. When these derangements occur, they should be corrected before surgical treatment. There was also prolonged preoperative hospital stay (8 days), which could be due to time needed to correct the fluid and electrolyte abnormalities, late admissions, unavailability of beds and busy surgical service.

In keeping with other studies [4, 19, 20, 27, 29], surgical intervention was the main stay of treatment performed in all of our patients. Open Ramstedt’s pyloromyotomy remains the standard procedure of choice for hypertrophic pyloric stenosis because it is easily performed and is associated with minimal complications. But since the 1990s laparoscopic pyloromyotomy is introduced and is said to have comparative outcome in terms of mortality and morbidity with superior cosmetic outcome [22–24]. Atropine has also shown some effect with prolonged treatment and can be an option for poor surgical candidates [30]. In this study, only open Ramstedt’s pyloromyotomy was done in all patients as there was no facility for laparoscopic pyloromyotomy at this centre.

Mucosal perforation is a rare intraoperative complication of Ramstedt’s pyloromyotomy and usually results from extending the myotomy beyond the pyloric–duodenal junction and is indicated by the appearance of bilious fluid. When this occurs, repair is done by using interrupted fine monofilament long-term absorbable sutures placed transversely and covered with omentum [31]. In our series, intraoperative mucosal perforations were reported in 5.9 % of cases, a figure which is higher than that reported in literature [26, 31, 32]. A high figure of 10.9 % was reported by Tadesse and Gadisa [29] in Ethiopia. This observation calls for meticulous care to be taken when performing Ramstedt’s pyloromyotomy to prevent mucosal perforation, especially at the lower end of the incision (pyloric–duodenal junction).

In the current study, the overall complication rate was 11.8 %, a figure which is high compared to what is reported in other studies [33, 34]. Surprisingly, the rate of surgical site infections in this study was significantly higher than that reported in literature [29, 33]. This finding calls for a need to identify factors responsible for this sad experience. In this study, all postoperative complications were treated conservatively except wound dehiscence in four patients which required surgical correction.

The median overall length of hospital stay in this study was 12 days, which was longer than most published data in literature [30, 34]. This could be due to prolonged preoperative hospital stay (8 days) which we recorded. The post-operative hospital stay was 5 days. However, due to the poor socio-economic conditions in Tanzania, the duration of inpatient stay for our patients may be longer than expected and this might have contributed to prolonged length of hospital stay in some of our patients.

Mortality after pyloromyotomy is less than 0.4 % in most major centers and when it occurs, it is usually from fluid and electrolyte depletion in infants presenting late, and inadequately corrected electrolyte problems before surgery [17, 18]. The overall mortality rate in this study was reported to be 4.9 % which is higher than 3.6 and 2.3 % reported in a Ghanaian and Iranian studies [35, 36], respectively. A high mortality rate of 6.7 % was reported by Caneiro [32] in Dar es Salaam, Tanzania. The high mortality rate in our study was attributed to age < 2 weeks, delayed presentation, severe dehydration on admission, hypokalaemia on admission and surgical site infection. Addressing these factors responsible for high mortality in our patients is mandatory to be able to reduce mortality associated with this disease. The fact that all deaths in our study occurred on the same postoperative day after a smooth operation raises a big question on our postoperative care both in terms of human power and facilities to care for postoperative infants who needs strict fluid balance, ambient environment to prevent hypothermia and other supportive cares. Such infants are transferred to the general pediatric surgical ward on the immediate postoperative period.

The potential limitation of this study is the fact that information about some patients was incomplete in view of the retrospective nature of the study. This might have introduced some bias in our findings. Poor documentation of data leading to exclusion of many patients was also a major limitation in this study. However, despite these limitations; findings from this study provide local data that can be utilized to improve the care of patients with IHPS in our local setting.

## Conclusion

This study has demonstrated that infantile hypertrophic pyloric stenosis is the most common cause of gastric outlet obstruction in infants in our setting. Age below 2 weeks, delayed presentation, prolonged preoperative hospital stay, surgical site infection and high proportion of dehydration and electrolyte disturbance were the main predictors of poor outcome. To avoid delay in diagnosis surgeons should have high index of suspicion in infants with non-bilious vomiting. There should be a guideline to correct the fluid and electrolyte disturbance after diagnosis to shorten preoperative hospital stay. Postoperatively; these patients have to be managed carefully in pediatrics ICU, at least for the immediate postoperative period. Further studies are needed in this area to improve for the care of infants with IHPS.
